# Toxicity effects of zinc supply on growth revealed by physiological and transcriptomic evidences in sweet potato (*Ipomoea batatas* (L.) Lam)

**DOI:** 10.1038/s41598-023-46504-2

**Published:** 2023-11-06

**Authors:** Yusha Meng, Chao Xiang, Jinxi Huo, Shengfa Shen, Yong Tang, Liehong Wu

**Affiliations:** 1 Institute of Crops and Nuclear Technology Utilization, Zhejiang Academy of Agricultural Sciences, Hangzhou, 310021 Zhejiang China; 2 Key Laboratory of Creative Agriculture, Ministry of Agriculture and Rural Affairs, Zhejiang Academy of Agricultural Sciences, Hangzhou, 310021 Zhejiang China

**Keywords:** Molecular biology, Plant sciences

## Abstract

Zinc toxicity affects crop productivity and threatens food security and human health worldwide. Unfortunately, the accumulation patterns of zinc and the harmful effects of excessive zinc on sweet potato have not been well explored. In the present research, two genotypes of sweet potato varieties with different accumulation patterns of zinc were selected to analyze the effects of excessive zinc on sweet potato via hydroponic and field cultivation experiments. The results indicated that the transfer coefficient was closely related to the zinc concentration in the storage roots of sweet potato. Excessive zinc inhibited the growth of sweet potato plants by causing imbalanced mineral concentrations, destroying the cellular structure and reducing photosynthesis. Furthermore, a total of 17,945 differentially expressed genes were identified in the two genotypes under zinc stress by transcriptomic analysis. Differentially expressed genes involved in the absorption and transport of zinc, defense networks and transcription factors played important roles in the response to zinc stress. In conclusion, this study provides a reference for the selection of sweet potato varieties in zinc contaminated soil and lays a foundation for investigating the tolerance of sweet potato to excessive zinc, which is meaningful for environmental safety and human health.

## Introduction

Zinc (Zn) is an imperative trace element required by plants and is involved in many metabolic and regulatory processes^1^. In nature, Zn generally exists in the form of divalent cations, participating in enzyme activity, gene expression, regulation, chromatin structure, and nucleic acid metabolism, among others^2^. Although Zn is an essential element, excessive exposure and ingestion of Zn is toxic to the human body, causing headaches, abdominal pain, nausea, and more^3^. In plants, Zn is closely interrelated with physiological, cellular, and molecular processes^4^. In particular, excessive Zn is toxic to most vascular plants and affects their growth and development^5^. Excessive Zn can be easily absorbed by plants and accumulates in the human body through soil-crop system and food chain, which makes Zn contamination an urgent issue to be resolved to ensure food safety worldwide^6^. Therefore, a better understanding of the accumulation patterns and toxic effects of Zn on crops is essential for human health.

Although Zn mainly accumulates in plant roots, the effects of Zn on the stem, leaves, and subcellular structure are essential for plants. Excessive Zn can damage plants by affecting growth and development, including seed germination and root and stem elongation, and even lead to death. This is due to Zn induced changes in enzyme activity, cytostructural disruption, and nitrogen metabolism imbalance^7–13^. Excessive zinc significantly reduces the fresh and dry weight of crops such as rice, maize, and *Triticum durum*, enhances antioxidant enzyme activity, increases antioxidant metabolites, reduces the relative growth rate and net photosynthesis, and even leads to leaf necrosis^7,14,15^. All these results suggest that excessive Zn has serious harmful effects on plants.

 Zn in roots is transported to aerial parts through both symplastic and apoplastic pathways in a Zn flux manner, and the transport mechanism varies among species and genotypes^2,16^. The *Km* values of Michaelis‒Menten functions for Zn uptake ranged from 0.6 to 2.3 nM in wheat plants^17^, whereas, the kinetics of Zn in influx per se did not play a significant role in Zn efficiency in wheat^17–19^. Differences in these parameters between Zn-efficient and Zn-inefficient genotypes of rice and tomato have been reported^20,21^. Sweet potato (*Ipomoea batatas* L., 2*n*=6*x*=90) is an important root crop worldwide, and it is the main food crop in many developing countries and regions^22,23^. It is rich in various nutrients such as vitamins and mineral elements, and plays an important role in ensuring human nutritional health. However, previous studies on Zn toxicity and tolerance mechanisms rarely focused on underground storage roots. Therefore, it is imperative to comprehensively understand the accumulation patterns and toxic effects of Zn on growth and development of sweet potato.

 In the present study, the accumulation pattern of Zn was investigated in two sweet potato varieties with different genotypes. The toxicity effects of Zn on sweet potato plants were evaluated under different concentrations of Zn. Subsequently, transcriptomic profiling of roots was conducted to comprehensively understand the mechanisms of toxicity effects of Zn on sweet potato. This research provides a reference for the selection of sweet potato varieties for Zn contaminated soil and lays a foundation for investigating the tolerance of sweet potato to excessive Zn.

## Results

### Zn accumulation patterns differ between the two sweet potato varieties

**Figure 1:**
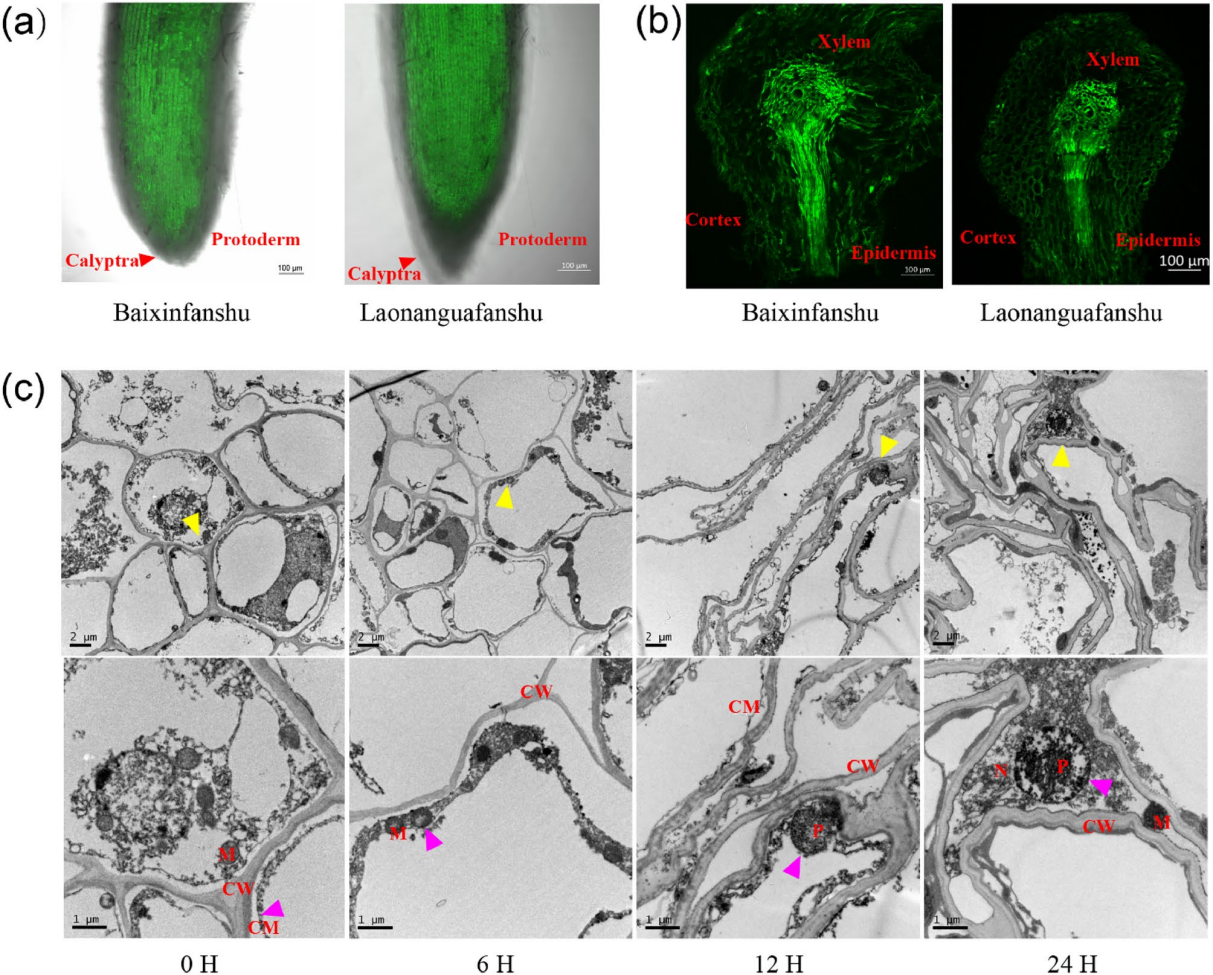
(**a**) Subcellular structure of sweet potato roots after 0 H, 6 H, 12 H, and 24 H exposure to 40 μM ZnSO_4_·7H_2_O, respectively. (**b**) Root longisection. (**c**) Root cross section. Note, Yellow arrows, magnified sites, purple arrows, damaged sites, red arrows, calyptra, CW, cell wall, CM, cell membrane, M, mitochondrion, N, nucleus, P, plasmosome.

Histochemical techniques for visualizing Zn showed that sweet potato roots absorbed and translocated Zn from roots to stems the via xylem (Fig. 1). Additionally, the protoderm of the calyptra in Baixinfanshu was thinner than that in Laonanguafanshu, and Zn staining in the xylem was brighter in Baixinfanshu (Fig. 1a,b). The thickness of the calyptra’s protoderm may be associated with the Zn absorption capacity of the two sweet potato varieties (Fig. 1a).

Table S1 showed that during the initial stage of storage root enlargement before harvest, Laonanguafanshu had a significantly higher Zn concentration than Baixinfanshu in storage roots. However, at harvest, the Zn concentration of storage roots in Baixinfanshu was significantly higher than that in Laonanguafanshu. At harvest, the Zn concentration in aerial parts decreased and the Zn concentration in storage roots increased (Table S1). These results indicated that in the later stage of storage root enlargement, more Zn was transported to the storage roots or that some of the aboveground Zn was redistributed to the storage roots to increase the Zn concentration of the storage roots.

**Figure 2:**
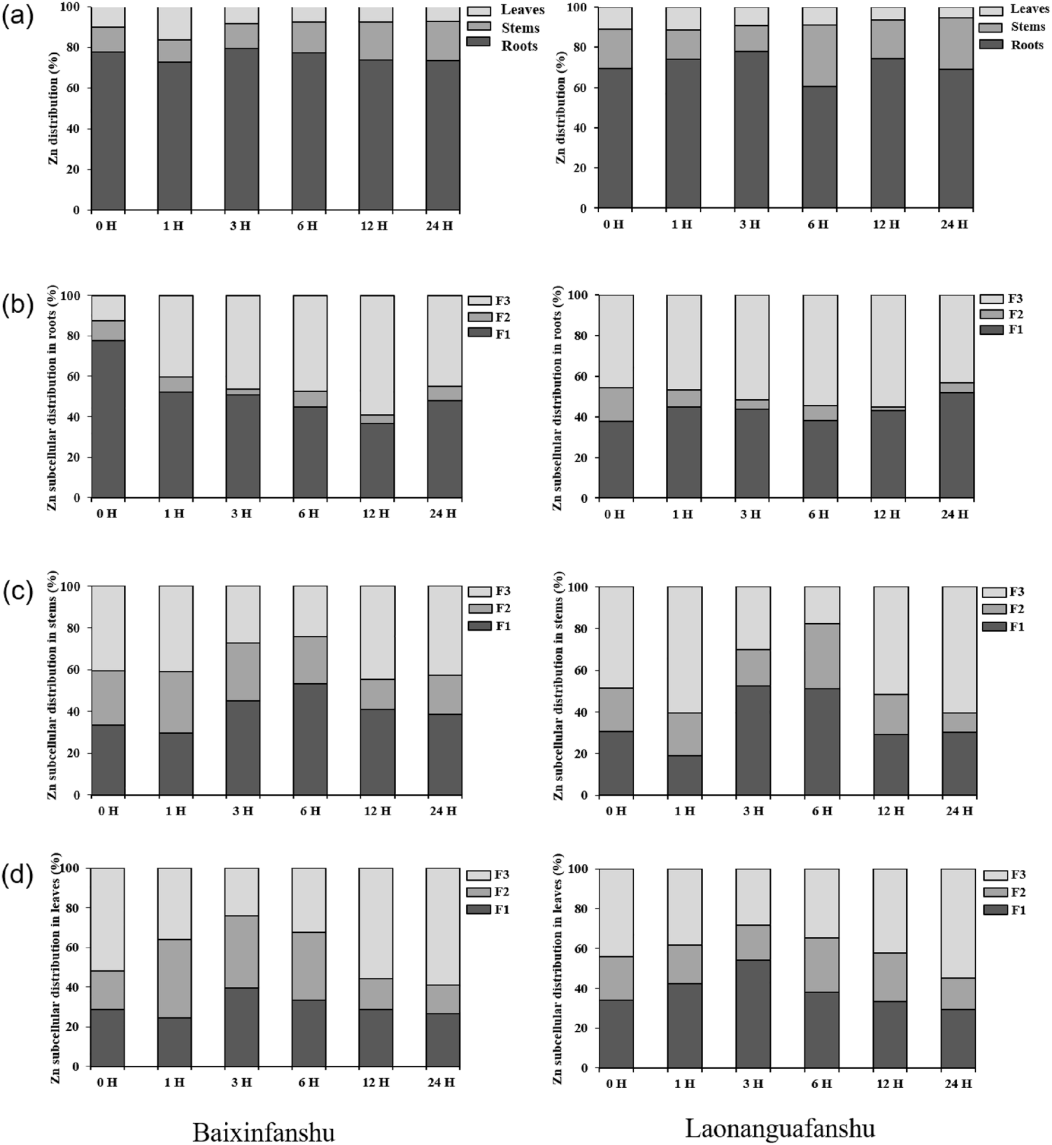
(**a**) Zn distribution in roots, stems and leaves of the two sweet potato varieties. (**b**) Subcellular distribution of Zn in roots. (**c**) Subcellular distribution of Zn in stems. (**d**) Subcellular distribution of Zn in leaves. In (**b**–**d**), F1, F2, and F3 represents the residue of Zn in cell wall, organelles, and soluble components (vacuoles), respectively. 0 H, 1 H, 3 H, 6 H, 12 H, and 24 H represent exposure time to 40 μM ZnSO_4_·7H_2_O (0 H was as the control). The results were showed as mean (n = 3).

The Zn distribution in roots, stems and leaves of sweet potato plants in the 12 treatment groups have been represented in Fig. 2a. Regardless of the applied treatment, Zn primarily accumulated in roots, and the accumulation pattern of Zn in plants was roots > stems >leaves. This may be the primary reason why the toxic effects of Zn on roots were more evident than those on aerial parts. The Zn concentration percentage in stems showed an upward trend in Baixinfanshu after 1 hour (Fig. 2a), while both varieties showed a decreasing trend in leaf Zn concentration. Root Zn concentration remained relatively stable despite the exposure time (Fig. 2a). These results suggested that excess Zn in the plant could move from root to stem once the Zn concentration reached saturation in the root.

The subcellular distribution of Zn in roots, stems, and leaves of the two varieties was significantly different (Fig. 2b–d). In stems and leaves, the proportion of Zn in organelles increased, while that in cell walls and vacuoles decreased over time (Fig. 2c–d). Zn accumulation in roots of Baixinfanshu mainly occurred in the cell walls (F1, 77.46%) in the control. Whereas, excess Zn was sequestered in the vacuoles (F3, 59.11%) to reduce cell damage (Fig. 2b) under Zn stress. Conversely, Zn accumulation in the cell walls and vacuoles in roots of Laonanguafanshu happened simultaneously. This may be one of the reasons for the different responses of Baixinfanshu and Laonanguafanshu to Zn stress.

### Zn inhibits the elongation of roots and damages the ultrastructure

The taproot and lateral root length decreased in both sweet potato varieties (10.85–34.99%), with the greatest inhibition occurring in Laonanguafanshu (34.99%) (Fig. S1a). The biomass of the roots was reduced in both varieties (41.7–47.86%), while the biomass of aerial parts was inhibited to a greater extent in Laonanguafanshu (20.63%) (Fig. S1c). Root surface area and root volume were also decreased in both varieties (17.03–30.19%) (Fig. S1c–d). Transmission electron microscopy (TEM) revealed that the ultrastructure of sweet potato roots was significantly influenced by 40 μM Zn^2+^, with cells becoming disorganized and organelles degrading over time (Fig. 1c). All the changes indicated that excess Zn inhibited root length, decreased biomass, damaged cell structure and even caused cell death in sweet potato roots.

### Zn stress causes mineral ions transfer disequilibrium

**Figure 3:**
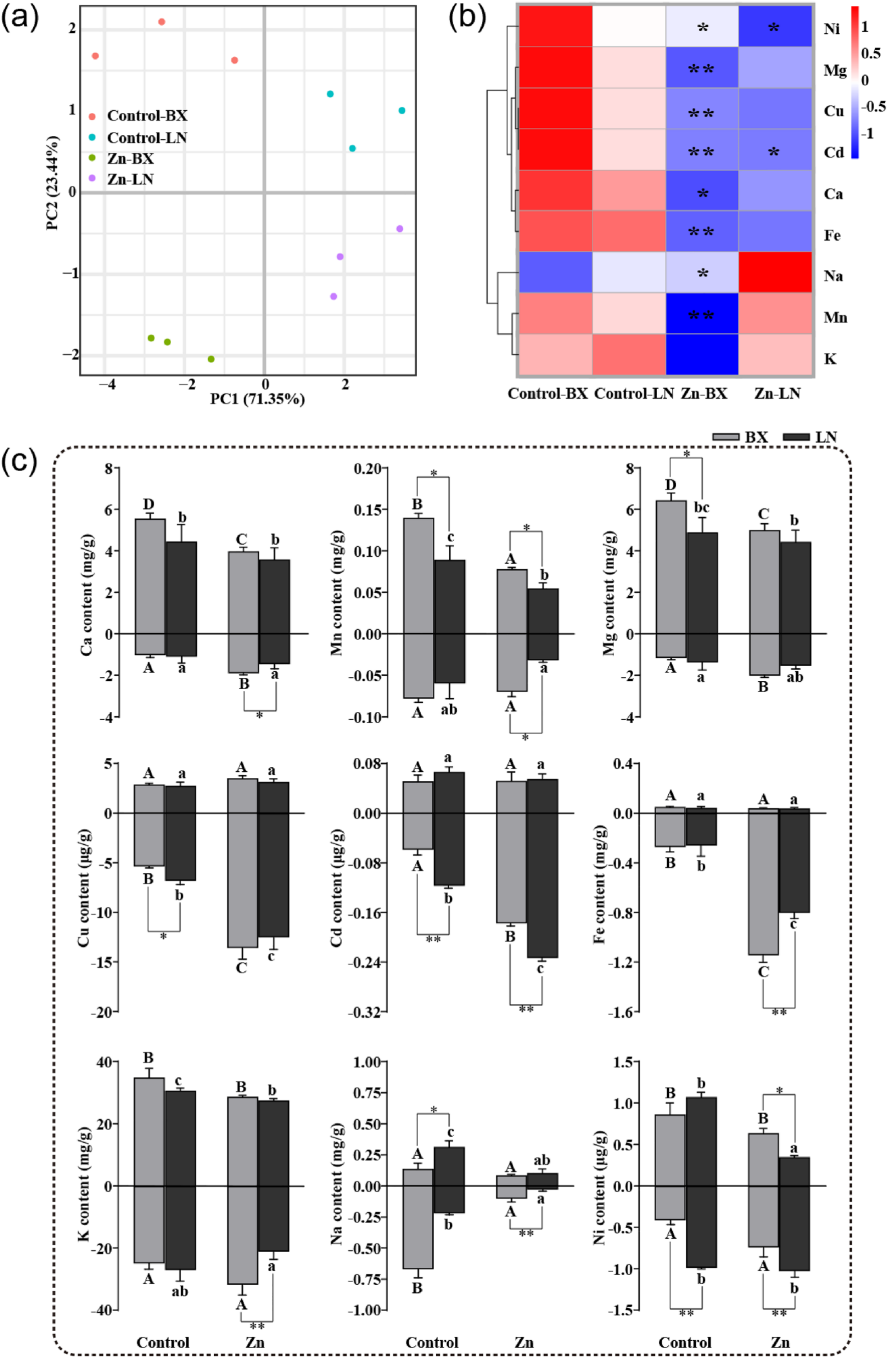
Effects of 40 μM ZnSO_4_·7H_2_O on mineral ions content. (**a**) PCA of mineral nutrition. (**b**) Heat map of TF in the two genotypes sweet potato plants. (**c**) Concentration of mineral elements in aerial parts and roots. BX, Baixinfanshu, LN, Laonangaufanshu. The lowercase letters indicated the significantly differences in content between the treatment and control in LN. The capital letters indicated the significantly differences in content between the treatment and control in BX. * and** indicated the significantly differences in content between BX and LN. The results were showed as mean ± SD (n = 3), (*P < 0.05, **P < 0.01, Duncan’s test).

The absorption of Zn, Ca, Mn, Cu, Fe, K and Na was significantly impacted by excessive Zn (Table 1, Fig. 3). Under natural conditions, Baixinfanshu had higher transfer factors than Laonanguafanshu, but these values plummeted under Zn stress. The PCA (principal component analysis, PCA) indicated that the mineral ions content in the two sweet potato varieties was significantly different under the control and Zn exposure conditions (Fig. 3a). According to the TF, only the transport levels of Ni and Cd were significantly inhibited in Laonanguafanshu, while the transport levels of most mineral elements were significantly inhibited in Baixinfanshu (Fig. 3b, Table 1). Ca, Fe, Mn, K, and Na concentrations were significantly higher in Baixinfanshu than in Laonanguafanshu (Fig. 3c). Ni and Cd concentrations were significantly lower in Baixinfanshu than in Laonanguafanshu. The concentrations of most ions in roots (Ca, Mg, Cu, Cd, and Fe) increased in both varieties compared to the controls (Fig. 3c). All these results suggest that Zn stress promoted or inhibited the absorption and transport some ions, resulting in ions disequilibrium in sweet potato plants.

**Table 1:** Uptake and transferring characteristics of Zn under hydroponic culture.

Variety	Treatment	Zn (mg kg^-1^ DW)		TF
		Aerial part	Root	
Baixinfanshu	Control	23.29 ± 4.06	26.03 ± 3.30	0.90^a^
	Zn (40 μM)	153.37 ± 8.54**	2143.41 ± 226.28**	0.07*
Laonanguafanshu	Control	18.36 ± 3.72	32.67 ± 6.84	0.59^b^
	Zn (40 μM)	202.83 ± 28.67*	3385.69 ± 440.85**	0.06*

The lowercase letters indicated the significantly differences in Zn content between the two genotypes sweet potato plants. * and** indicated the significantly differences between Zn treatment and the control. The results were showed as mean ± SD (n = 3), (*P < 0.05, **P < 0.01, Duncan’s test).

### Photosynthetic parameters and proline content are significantly affected by Zn stress

**Figure 4:**
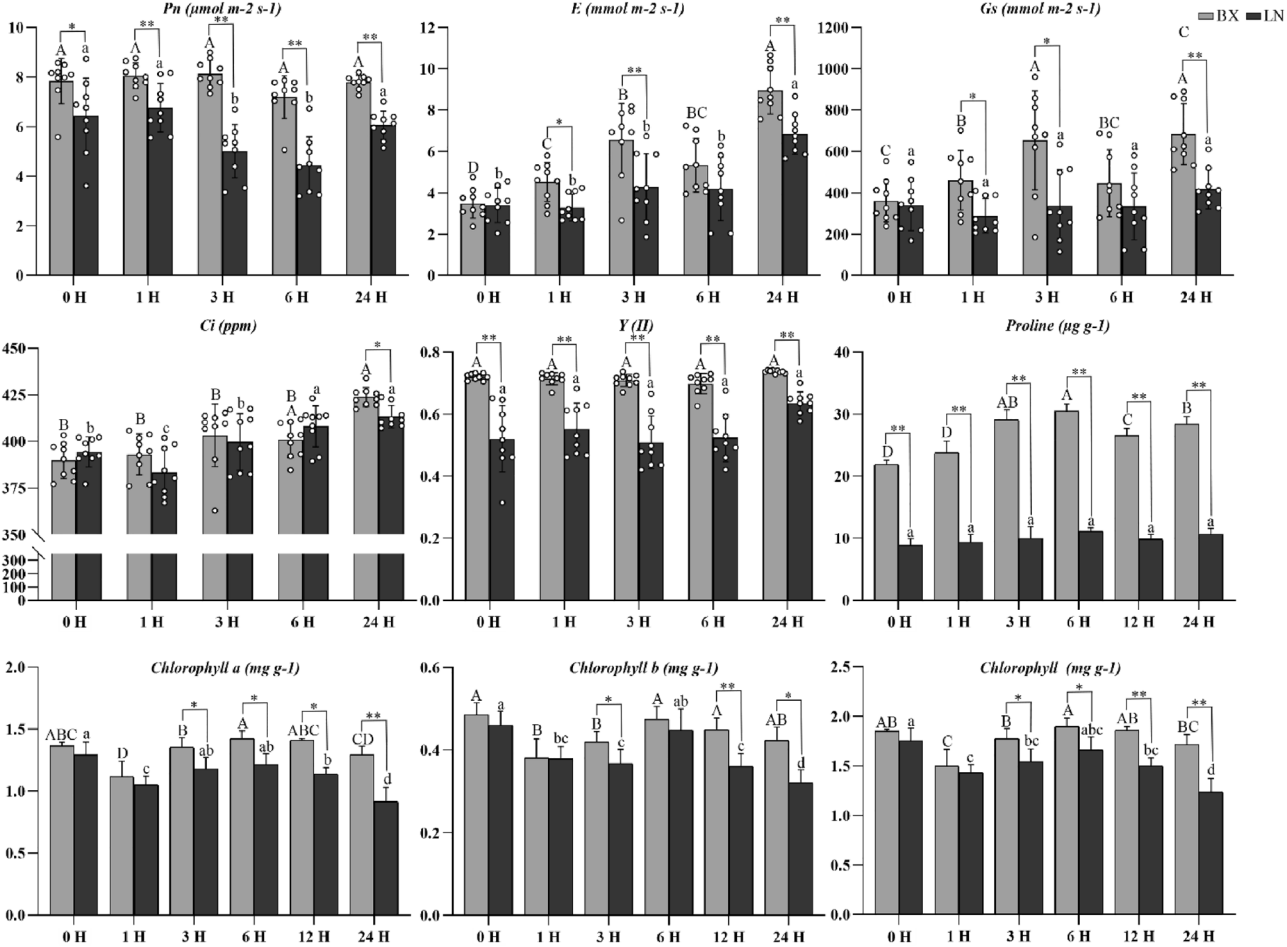
Effects of 40 μM ZnSO_4_·7H_2_O on photosynthetic, chlorophyll and proline contents. Pn, E, Gs, Ci, and Y(II) represents net photosynthetic rate (μmol m^-2^ s^-1^), transpiration rate (mmol m^-2^ s^-1^), stomatal conductance (mmol m^-2^ s^-1^), intercellular carbon dioxide concentration (ppm), and photochemical quantum yield of photosystem II. BX, Baixinfanshu, LN, Laonangaufanshu, 0 H, 1 H, 3 H, 6 H, 12 H, and 24 H represent exposure time to 40 μM ZnSO_4_·7H_2_O (0 H was as the control). The lowercase letters indicated the significantly differences in content between the treatment and control in LN. The capital letters indicated the significantly differences in content between the treatment and control in BX. * and** indicated the significantly differences between BX and LN. The results were showed as mean ± SD (n ≥ 3), (*P < 0.05, **P < 0.01, Duncan’s test).

Pn was significantly reduced by 22.24% and 30.95% in 3 hours and 6 hours, respectively, in Laonanguafanshu (Fig. 4). However, there was no significant change in Pn of Baixinfanshu. In Baixinfanshu, the levels of E and Gs were significantly increased by 157.33% (0 H vs. 24 H) and 89.69% (0 H vs. 24 H), respectively (Fig. 4). E was significantly increased by 105.91% (0 H vs. 24 H) in Laonanguafanshu (Fig. 4). These parameter changes may be caused by inhibited photosynthesis and increased respiration. Y(II) showed no significant change in either variety (Fig. 4). The content of chlorophyll a and chlorophyll b significantly decreased in both varieties, but was more severe in Laonanguafanshu (Fig. 4). This may be responsible for the reduced photosynthesis in this variety. The proline concentration significantly increased in the Baixinfanshu treatment plants, but there was no significant change in the proline concentration of Laonanguafanshu (Fig. 4). These findings indicated that there were significant differences in photosynthesis and proline accumulation in response to Zn stress between the two genotypes.

### Genes involved in absorption and transport of Zn are reprogrammed under Zn stress

To comprehensively study the transcriptome related to Zn stress in sweet potato, 36 RNA libraries were sequenced, with raw reads ranging from 42.14 to 47.12 million. Low-quality reads were discarded, and the clean reads were then aligned to the reference sweet potato genome database. In total, 1,604,519,946 clean reads were obtained from the 36 RNA-seq libraries, with a mapped read percentage of 71–79%.

**Figure 5:**
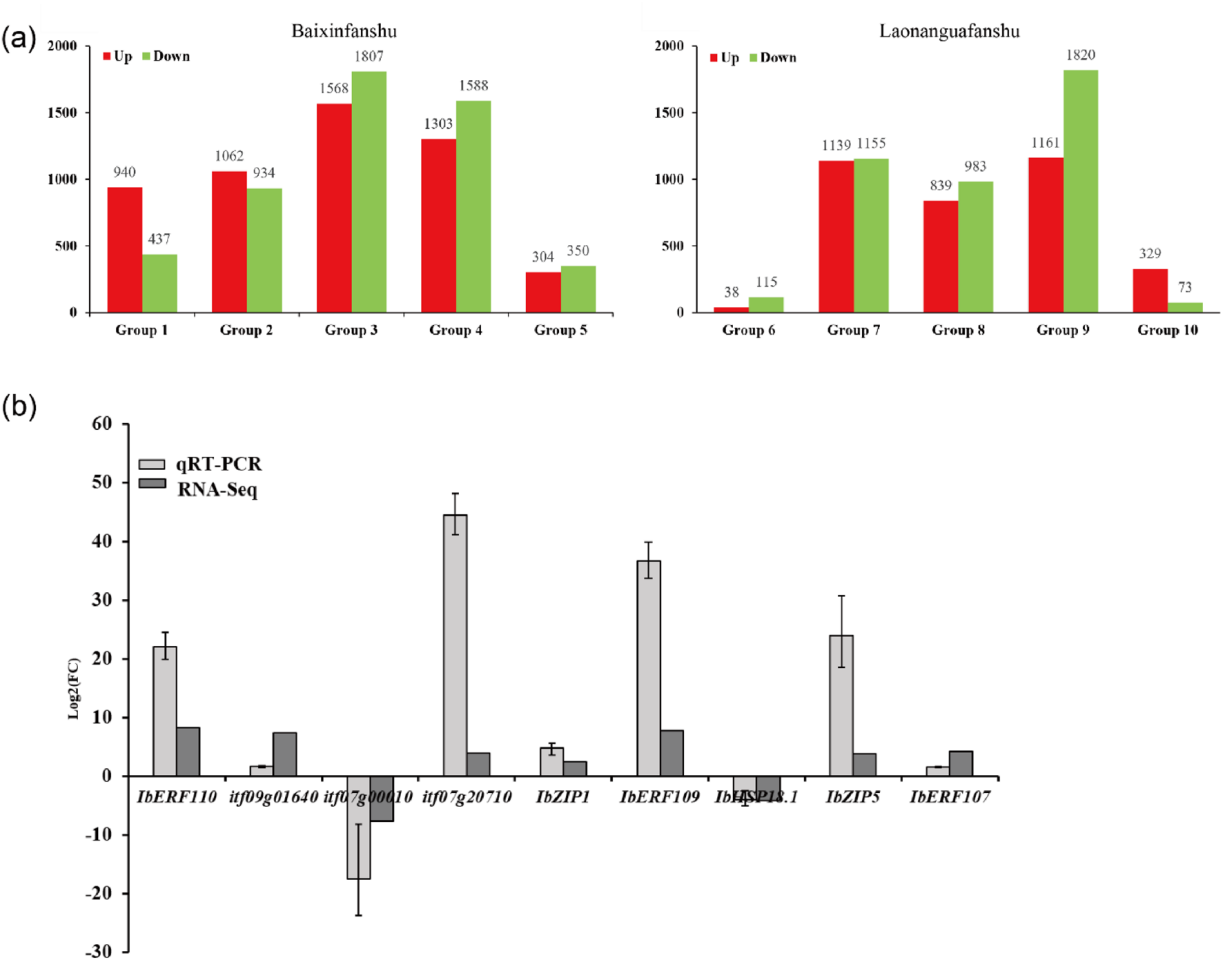
(**a**) Number of significantly differentially expressed genes. (**b**) Comparison of gene fold changes (relative to the control) between qPCR and RNA-Seq results. In (**a**), red indicated significantly up-regulated, green indicated significantly down-regulated genes. *P* < 0.05 and |Log2(foldChange)|> 1 represented significantly DEGs. The results were showed as mean ± SD (n = 3). Three independent biological replicates were performed.

DEGs of the two sweet potato plants were analyzed via pairwise comparisons in 10 groups (BX 1 h, BX 3 h, BX 6 h, BX 12 h and BX 24 h vs. BX 0 h, LN 1 h, LN 3 h, LN 6 h, LN 12 h and LN 24 h vs. LN 0 h). The total number of DEGs was 10293 and 7652 in Baixinfanshu and Laonanguafanshu, respectively (Fig. 5a). We also observed that the number of up- and down-regulated genes peaked in BX at 6 h, but peaked in LN at 12 h (Fig. 5a). These findings indicated that the response degree and response time were different between the two genotypes under Zn stress. Venn diagrams revealed that the DEG distributions were different across both groups and varieties (Fig. S2a). Baixinfanshu had 386, 216, 822, 695 and 218 DEGs unique to each library, while Laonanguafanshu had 30, 796, 341, 1348, and 60 DEGs unique to each library. A total of 103 and 18 DEGs were shared among the 5 libraries in Baixinfanshu and Laonanguafanshu, respectively (Figure S2a). Among the DEGs, 63 transporters were identified. These transporters belong to five known Zn transporter families, of which the ZIP gene family has the most members (Table 2, Table S2). Importantly, there were 12 and 18 upregulated transporters in Baixinfanshu and Laonanguafanshu, respectively. However, there were 43 and 37 downregulated transporters in Baixinfanshu and Laonanguafanshu, respectively (Table 2, Table S2). This indicated that most Zn-related transporters were downregulated under Zn stress to restrain Zn absorption and translocation in plants and protecting plants from excessive Zn. These results suggested that the expression levels of genes involved in Zn uptake and transfer were reprogrammed under prolonged stress time.

**Table 2:** DEGs related to 5 known families of Zn transporters.

Zn transporter family	Total members	Baixinfanshu		Laonanguafanshu	
		Up	Down	Up	Down
ZIP gene family	37	5	28	6	29
Natural resistance-associated macrophage protein	1	–	1	–	1
Major facilitator superfamily	22	7	13	11	5
P-type ATP-ase	1	–	–	–	1
Metallothionein, family	2	–	1	1	1
Total	63	12	43	18	37

**Figure 6:**
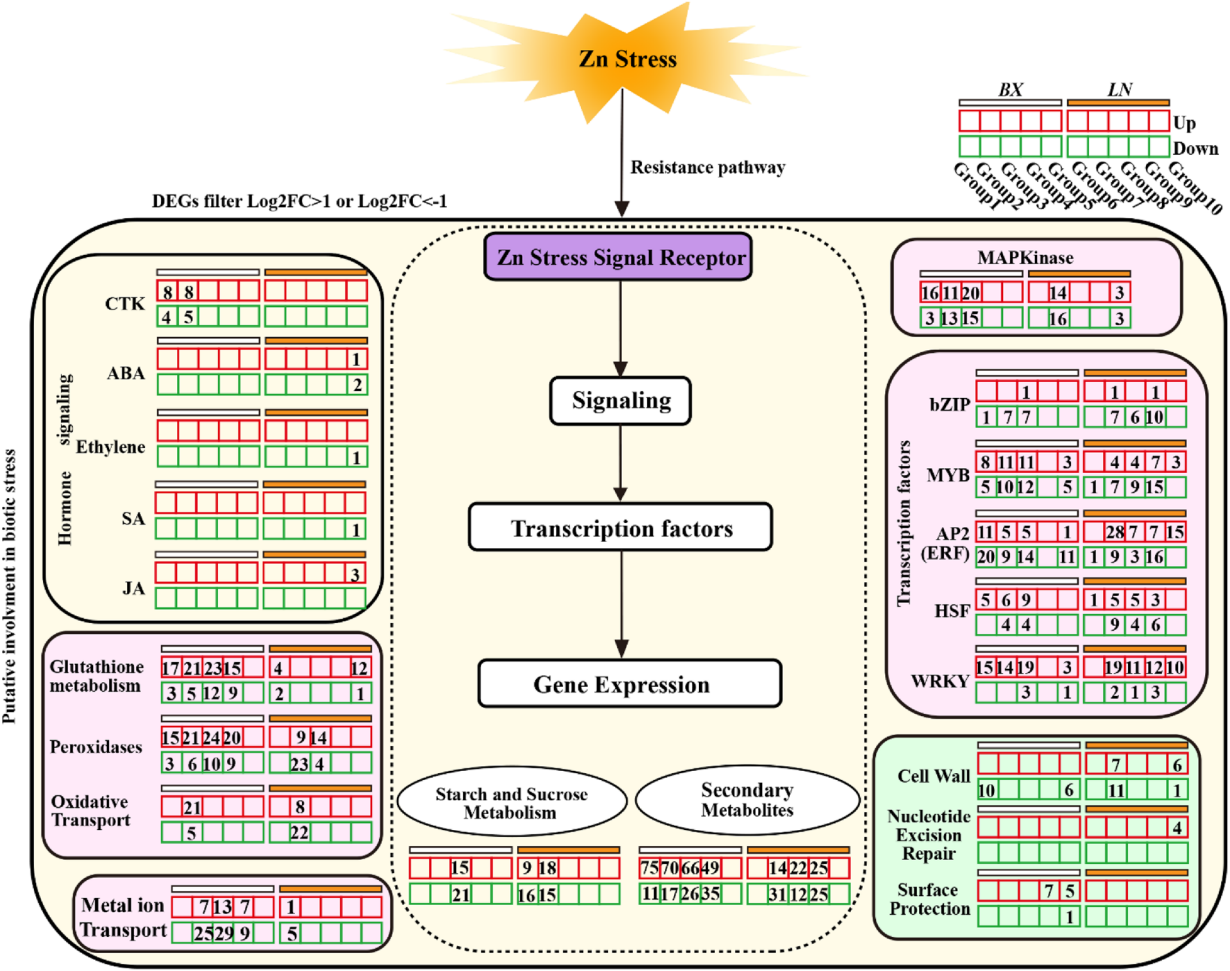
The induced defense network by Zn toxicity in sweet potato roots. The red and green grids represented up-regulated and down-regulated pathways, respectively. BX, Baixinfanshu, LN, Laonangaufanshu.

In addition, Zn stress significantly changed the expression levels of defense-related genes, and various pathways participated in the defense network with the extension of exposure time (Figure 6, Figure S2b,c). For instance, the MAPK signaling pathway-, GSH metabolism-, phenylpropanoid biosynthesis-, peroxidase-, oxidative stress response-, and starch and source metabolism-related genes were abnormally expressed (Fig. 6, Fig. S2b,c). Notably, the expression of genes related to GSH metabolism, peroxidase activity, the MAPK signaling pathway, and secondary metabolites was significantly more highly induced in Baixinfanshu than in Laonanguafanshu (Fig. 6, Fig. S2b,c). More interestingly, MYB, ERF, HSF and WRKY transcription factors were more upregulated in Baixinfanshu than in Laonanguafanshu at 1 and 3 hours, whereas the situation was reversed at 12 and 24 hours (Fig. 6). This may indicate that there is a faster or more sensitive Zn stress defense mechanism in Baixinfanshu than in Laonanguafanshu. Surface protection (cutin, suberin and biosynthesis) was only identified in Baixinfanshu. However, nucleotide excision repair was only identified in Laonanguafanshu in the later stage (Fig. 6). Genes related to metal ion transport exhibited a more active mode of expression in Baixinfanshu (Fig.6), which may be another factor affecting the absorption and translocation of Zn in sweet potato plants.

To verify the reliability of the transcriptomic data, ten DEGs including Zn transport genes (*IbZIP1*, *IbZIP5*), ethylene-responsive transcription factors (*IbERF107*, *IbERF109*, *IbERF110*), hydrolases (itf07g20710, itf09g01640, itf07g00010), heat shock protein (IbHSP18.1) and U-box protein 21 (itf14g00200) were selected for qRT‒PCR. The relative expression trends of these 10 genes were highly consistent with the RNA-seq results (Fig. 5b). The above results indicated that transporters and transcription factors involved in absorption and transport of Zn played important roles in defense network under Zn stress in sweet potato.

## Discussion

Zn is absorbed by root epidermis. This study demonstrated a working model of Zn accumulation in sweet potato plants. The thickness of the protoderm of the calyptra between the two varieties was different (Fig. 1a). There have been many studies on the absorption and transportation of ions in plants root^16^, and based on these studies, we speculated that the thickness of the calyptra may affect the absorption of Zn. The thinner the protoderm of the calyptra is, the higher the transfer coefficient (Table 1, Fig. 3). Zn is transported to cortical cells and then to stems through xylem. During this transportation, Zn fluxes flow toward the aerial parts of plants by symplastic and apoplastic pathways^16^. The accumulation of excess Zn in this study was mainly in cell wall and vacuoles of root cells (Fig. 2b). Similar results have been reported by Kaur and Garg^5^. We also investigated the subcellular distribution of excess Zn in stems and leaves in sweet potato plants, and the results were similar to those in roots. Surprisingly, the proportion of Zn in stem and leaf organelles (F2) was higher than that in roots, especially that in leaves (Fig. 2c,d). We speculated that this may be because photosynthesis takes place in stems and leaves, and Zn is the activator of many key enzymes in this process. Küpper et al. reported similar results in *Arabidopsis helleri* and *Noccaea caerulescens*^24^.

Islam et al. reported that the exposure of maize plants to 50 µM Zn inhibited biomass production, decreased chlorophyll and total soluble protein and strongly increased the accumulation of Zn in both roots and shoots^7^. In durum wheat seedlings, excess Zn inhibited the relative growth rate (RGR), retarded CO_2_ assimilation rates (approximately 30%) in the second developed leaf, and disturbed photosynthetic electron transport processes^15^. The toxicity of excessive Zn has also long been recognized in *Oryza sativa*^13,25^. In this study, high amounts of Zn significantly inhibited root length and biomass (Fig. S1). The cell structure was destroyed or cell death occurred when cells were exposed to Zn stress (Fig. 1c). All experiments in the present study clearly showed that excessive Zn significantly inhibited primary root elongation, decreased biomass, and retarded photosynthesis in sweet potato plants.

Researchers have reported that high Zn levels hinder not only ions (N, P, K, Mg, Ca, Fe, etc.) uptake but also ions allocation to different plant parts^8,26^. Souza et al. also found that differential alterations in nutrient absorption and distribution lead to an increase in some nutrients and a decrease in others, depending on the species/organs of plants^27^. In the present research, the concentration of elements, including Zn, was also out of balance (Fig. 3, Table 1). The K^+^/Na^+^ equilibrium, which could regulate osmotic stress, was disrupted in roots as well as aerial parts (Fig. 3c). Zn shares absorption and transport pathways with other mineral elements^28^. In both varieties, the TF of Zn significantly decreased under Zn exposure (Table 1), indicating that Zn absorption and transport were inhibited. Although the TF of Fe was also significantly inhibited by Zn, the Fe concentration was significantly higher than that in the control (Fig. 3b,c). This may be caused by the transcriptional response of Fe-regulated genes stimulated by high Zn. Consistent with previous studies, high Zn levels interferes with mineral ions balance in sweet potato plants^8,26,27^. Chen et al. also found that Cd TF significantly affected the Cd concentration in rice grains^29^. The Zn concentration was low in Baixinfanshu at the initial stage of storage root enlargement (Table S1). At the harvest stage, the Zn concentration was higher in Baixinfanshu than in Laonanguafanshu (Table S1). The Zn content of storage roots comes from two sources: the soil and redistribution from the aerial parts^30^. Therefore, we speculated that during the harvest stage, Baixinfanshu with high Zn TF leads to more Zn being transported from the soil and aerial parts to the storage roots. Some sweet potato varieties with low Zn TF have the potential to be planted in Zn contaminated soil and have safe Zn concentrations in edible storage roots. This is very important for ensuring food safety.

In *Polypogon monspeliensis*, competitive dislocation of Zn to Mn during the photolysis of water has an effect on PSII photochemistry and hinders electron transport and O_2_ evolution^31^. Mn decreased in aerial parts and roots in this study, and we speculated that it may be related to the decrease in Pn (Figs. 3c, 4). Similar to the research in *Cajanus cajan L. Millsp.*^8^, the chlorophyll a and chlorophyll b contents in sweet potato also decreased significantly under Zn stress (Fig. 4). A study in *Z. fabago* found that the quantum yield of electron flow through PSII (ΔFv/F’m) decreased significantly under 50 μM Zn stress^32^, but there was no significant change in this study (Fig. 4). Proline, a low-molecular-weight chaperone, is an important stress metabolite with essential functions in osmotic adjustment and survival of plants under Zn-stressed conditions^33,34^. The proline content was different between the two varieties (Fig. 4). These results may be related to the species and Zn concentration. Therefore, it can be inferred that Zn stress does not have a single specific photosynthetic target in sweet potato plants. In contrast, the toxicity of Zn may trigger a cascade of abnormal reactions.

In the present research, we also obtained a high-quality sweet potato root unigene database through RNA-seq (Fig. S2). High Zn stimulated the transcriptional response of many Zn-regulated genes at the RNA level. The cell wall pathway, secondary metabolite pathway, MAPK signaling pathway, peroxidase activity, and GSH all responded to Zn toxicity, but the reaction time and degree were different in the two varieties (Fig. 6).

The cell wall, as the primary barrier against stress, plays an important role in stress defense. Lignin is the main component of the secondary wall, it forms an interlacing network to harden the cell wall and consists of many highly branched polymers of phenylpropanoid groups, which is consistent with pathway analysis (Fig. S2). Moreover, genes related to xyloglucan endotransglucosylase, which can hydrolyze pectin in the cell wall, were down-regulated to protect the cell wall (Fig. S2cTherefore, pectin binds to superfluous Zn in the cell wall, and callose deposits hold it in place to ensure that Zn cannot enter the cytoplasm, but this may cause growth inhibition^35^. Dai et al. discovered similar results in mulberry under Cd stress^36^.

It has been well documented that secondary metabolites play an important role in combating stress^37^. Secondary metabolites (polyphenols, terpenes, and vitamins) can combat reactive oxygen species (ROS) and help prevent oxidative stress^38^. In this study, the contents of all these metabolites were significantly increased (Fig. 6). Free amino acids and soluble sugars play an essential role in intracellular osmoregulation, and sugars can also remove ROS and induce the expression of antioxidant-related genes in plants^34^.

MAPKs are one of the most studied signaling mechanisms in plants and are involved in various signal transduction pathways (pathogens, drought, salinity, cold, trauma, ozone, ROS, and hormone stimuli)^39^. The DEG results indicated that these responsive genes related to Zn toxicity differed between the two varieties. According to Lai et al. and this study, MAPKs are also involved in heavy metal stress (Fig. 5)^40^. GSH helps eliminate excess cytoplasmic Zn by forming complexes with Zn^2+^, thereby reducing toxicity^5^. Organic compound transporters are responsible for transporting sugars, amino acids and organic acids.

Five known families (ZIP transporters, major facilitator superfamily, p-type ATPase, natural resistance-associated macrophage protein and metallothionein family) have been widely documented to promote Zn transport and control Zn homeostasis in plants^30,41^. A total of 63 significantly differentially expressed genes belonging to the 5 known families were identified in this study (Table 2, Table S2). The ZIP protein family plays an important role in the absorption and transport of cations such as Zn, and 15 *ZIP* genes have been identified in *Arabidopsis thaliana*^42–44^. Here we found 4 differentially expressed *IbZIPs* in response to Zn, indicating that they played important roles in Zn detoxification in sweet potato (Table 2, Table S2). Based on these molecular data, we hypothesized that the tolerance of sweet potato to Zn is achieved by enhancing the cytoplasmic accumulation of Zn, producing more soluble content, and reducing the absorption and transportation of Zn in cells. In addition, transcription factors such as bZIP, MYB, AP2, HSF, and WRKY were all stimulated by Zn stress. These transcription factors may play key roles in the regulation of Zn transport genes.

## Materials and Methods

### Plant materials and treatments

The two sweet potato cultivars used were Baixinfanshu and Laonanguafanshu, which are two genetically different landraces preserved at the Sweet Potato Germplasm Repository at the Institute of Crop and Nuclear Technology Utilization, Zhejiang Academy of Agricultural Sciences, Hangzhou, China. The zinc concentration in the storage roots of Baixinfanshu is high, while it is low in Laonanguafanshu. The virus-free seedlings of Baixinfanshu and Laonanguafanshu were cultivated in test fields and an artificial climate chamber at Zhejiang Academy of Agricultural Sciences. For field cultivation, plants were transplanted to the field in May of 2019 and 2020, with only organic manure as the base fertilizer and normal field management. Twenty plants were used as an experimental plot, and three replicates were set, with two years of repeated experiments. For the hydroponic experiments, 10 cm long sweet potato stems were cut and inserted into plastic hydroponic tanks and fixed with foam, and each hydroponic tank contained four plants. The plants were cultured in half-strength Hoagland solution until new roots emerged (approximately 2 weeks) in an artificial climate chamber with a temperature of 25/20 °C (light/dark), photoperiod of 12/12 h (light/dark) and relative humidity of approximately 75%. The nutrient solution was replaced every 3 days.

Based on previous studies, the concentrations of Zn^2+^ added under hydroponics ranged from 0 to 200 μM^45,46^. Therefore, a hydroponic experiment was conducted to examine the effects of Zn^2+^ (supplied as ZnSO_4_·7H_2_O) at different levels (0, 0.4, 4, 40, 100 and 200 μM) on sweet potato plants. When the addition amount was 40 μM Zn^2+^, the growth inhibition effect was significant. Therefore, the addition level of Zn^2+^ in this study was 40 μM (Fig. S3).

### Zn uptake, translocation, and allocation within sweet potato plants

#### Root microstructure analysis

Sterile Baixinfanshu and Laonanguafanshu plants were prepared and cultured in half-strength Murashige & Skoog (MS) medium until new roots developed. The distribution and accumulation of Zn in sweet potato roots were investigated according to Seregin et al. by confocal laser scanning microscopy (Zeiss LSM880 with Airyscan)^47^.

#### Zn distribution in sweet potato plants and subcellular localization

The two varieties were planted in a test field without any pollution, with organic manure as the base fertilizer only and normal field management. Samples of Baixinfanshu and Laonanguafanshu plants (75 d and 135 d, respectively) growing in the field were taken and cleaned three times in deionized water, divided into aerial parts and storage roots, and finally cut into strips. All the samples were baked in an oven to constant weight at 75 °C, ground into powder by a machine, and sifted through a 100-mesh sieve to remove residues. Taking 0.1 g of grinding powder from each sample into the digestive tube, 1 ml hydrogen peroxide and 5 ml nitric acid were added for half an hour of predigestion, and then digested in a microwave reaction system (CEM Mars 6). Finally, the volume was fixed to 20 ml. The Zn concentrations in the aerial parts and storage roots were determined by inductively coupled plasma‒mass spectrometry (ICP‒MS) (Analytik Jena AG, Germany).

After culturing for 2 weeks in half-strength Hoagland solution, cuttings were treated with 40 μM Zn^2+^ solution for 0 (as a control), 1, 3, 6, 12, and 24 h in an artificial climate chamber. To determine the Zn distribution in sweet potato plants, the harvested plants were washed in deionized water three times, rinsed three times in 0.5 mM CaCl_2_ to remove the Zn^2+^ adsorbed on the root surfaces, and again washed in deionized water three times. The cleaned samples were divided into roots, stems, and leaves, and then baked, ground, sifted and digested as described above.

To determine the Zn distribution in subcellular compartments, another three fresh and ablated roots, stems, and leaves were processed at 4℃ as follows: 10 ml of grinding buffer (50 mM Tris–HCl buffer solution of pH 7.5 with 0.25 mM sucrose and 1 mM β-mercaptoethanol) was added to 2.0 g fresh tissue for complete grinding, the homogenate was centrifuged at 1000 × g for 15 min, and the cell wall component (F1) was collected from the precipitate. The supernatants were transferred to a new centrifuge tube and centrifuged at 10,000 × g for 45 min, and the organelle-containing component (F2, nucleus, mitochondria, and more) were collected from the precipitate. The final supernatant was the soluble component (F3, mainly the cytoplasmic matrix component)^40^. All the precipitates and supernatants of each sample were dried, digested, and determined as described above. Three independent biological replicates were performed.

### Effects on sweet potato plants

#### Effects on plant growth and mineral ions content

After culturing for 2 weeks in half-strength Hoagland solution, cuttings were treated with 40 μM Zn^2+^ solution once every three days for 10 days in an artificial climate chamber. The sampled plants were washed as described and divided into two equal parts, one to determine the effect of Zn on growth and the other to determine the effect of Zn on mineral ion content. For growth determination, the length of the primary root and lateral root, surface area, and volume of roots were determined using a ruler and WinRHIZO system for each sample. For biomass assays, samples were oven-dried for five days to a constant weight at 75 °C and weighed by a precision electronic auto balance. For mineral ions content determination, the samples were washed, divided into aerial parts and roots, baked, ground, and sifted as described above. A 0.1 g sample of grind powder from each sample was added into a digestive tube, predigested and digested as described above. The Zn, Ca, Mn, Mg, Fe, Cu, Ni, and Cd concentrations were determined by ICP‒MS (Analytik Jena AG, Germany). The K and Na concentrations were determined by a graphite furnace atomic absorption spectrometer (GFAAS) (ZEETnit700P, Analytik Jena AG, Germany). Three independent biological replicates were performed.

#### Effects on root ultrastructure

After culturing for 2 weeks in half-strength Hoagland solution, cuttings were treated with 40 μM Zn^2+^ solution. At 0 (as a control), 1, 3, 6, 12, and 24 h of treatment, the roots mature zone were excised and immediately fixed in fixative solution and prepared for transmission electron microscopy (TEM, Hitachi H7650) according to Lai et al. to determine the effects of excess Zn on root ultrastructure^48^. Three independent biological replicates were performed.

#### Effects on photosynthetic, chlorophyll and proline contents

After culturing for 2 weeks in half-strength Hoagland solution, cuttings were treated with 40 μM Zn^2+^ solution. At 0 (as a control), 1, 3, 6, 12, and 24 h of Zn treatment, the photosynthetic parameters were measured by a GFS-3000 (Heinz Walz GmbH, Germany). The photosynthetic parameters included Pn (photosynthesis rate), E (transpiration rate), Ci (internal CO_2_ concentration), Gs (stomatal conductance) and Y(II) (actual photochemical efficiency of PSII). The contents of chlorophyll a and chlorophyll b were measured as described by Zhang et al.^49^. Proline content was measured with assay kits (Nanjing Jiancheng Bioengineering Institute, Nanjing, China). Three independent biological replicates were performed.

### RNA sequencing and data analysis

After culturing for 2 weeks in half-strength Hoagland solution, cuttings were treated with 40 μM Zn^2+^ solution for 0 (as a control), 1, 3, 6, 12, and 24 h. The sampled roots were used for total RNA extraction according to the RNAprep Pure Plant Plus Kit (Tiangen Biotech, Germany). cDNA libraries were constructed and sequenced on an Illumina NovaSeq 6000 platform (Novogene Co. Ltd, Beijing, China). Three independent biological replicates were performed. In-house Perl scripts were used to preprocess the raw reads for quality, and then the clean reads were mapped to the sweet potato reference genome^50^. Differentially expressed genes (DEGs) were identified using DESeq2 in the R platform (1.20.0). Genes with an adjusted *P* value ≤ 0.05 as well as a |log2FoldChange|> = 1.0 were deemed significantly differentially expressed. GO (Gene Ontology) enrichment analysis and Kyoto Encyclopedia of Genes and Genomes (KEGG) pathway annotations were performed by the clusterProfiler R package with a corrected *P* value cutoff of 0.05.

### qRT‒PCR analysis of ten DEGs

The treated seedling roots (0 h as a control, and 1-, 3-, 6-, 12- and 24-h exposures to 40 μM Zn^2+^ were sampled for total RNA extraction using TRIzol reagent. The cDNA was obtained according to the PrimeScript™ RT reagent Kit with gDNA Eraser (TaKaRa Biomedical Technology Co., Ltd, Beijing, China). Ten DEGs (including Zn transporters, transcription factors, hydrolase, and proteins) were selected for qRT‒PCR in both genotypes. TB Green-based reactions were conducted on Bio-Rad CFX96 platform according to the amplification process: 94 °C for 30 s followed by 40 cycles at 94 °C for 5 s, 60 °C for 30 s, and 72 °C for 10 s. The *Actin* (AY905538) gene of sweet potato was selected as an internal control^51^. The expression level of each selected gene was calculated by the 2^−ΔΔCt^ method^52^. The experiments were conducted with three biological replicates.

### Statistical analysis

To study the absorption and transport capacity of Zn^2+^ in sweet potato, the transfer coefficient (TF) was analyzed according to the formula presented by Chen et al.^53^. All data presented in this study are the mean values of at least three biological replicates. One-way analysis of variance (ANOVA) followed by Duncan’s test was used to compare the difference between the control and treatments (*P < 0.05, **P < 0.01, SPSS statistical software package version 16.0 (IBM Corp., Armonk, NY, USA).

$$Transfer\,coefficient\,of\,Zn \left(TF\right)=\frac{[element\,concentrations]\,aerial\,parts}{[element\,concentrations]\,roots}$$

## Conclusions

Field and hydroponic studies were conducted on two sweet potato varieties with differences in Zn accumulation, elucidating a relatively conserved physiological and molecular mechanism of Zn absorption, transport, accumulation, toxicity and tolerance in sweet potato. High levels of Zn significantly inhibited the elongation of the primary root, disturbed the balance of mineral ions metabolism, reduced photosynthesis, and decreased biomass in sweet potato plants. The accumulation pattern of Zn was as follows: roots > stems > leaves. The root cell walls and vacuoles were the main sites of Zn subcellular distribution. Zn and other mineral elements may share pathways for entering the root. Transcriptomics analysis showed that Zn toxicity significantly affected the expression of genes associated with Zn transporters and stress-related pathways. In conclusion, the present results lay a foundation for investigating the tolerance of sweet potato to excessive zinc. There is a close relationship between TF and Zn concentration in storage roots of sweet potato. Some sweet potato varieties with low Zn TF have the potential to be planted in Zn contaminated soil, which is very important for ensuring global food security and human health.

## Data availability

The data presented in this study are available on request from the corresponding author.

### Supplementary Information

Supplementary Information.
