# Supplementary material for: Toxicity effects of zinc supply on growth revealed by physiological and transcriptomic evidences in sweet potato (Ipomoea batatas (L.) Lam)
Source: Sci Rep. 2023 Nov 6;13:19203. doi: 10.1038/s41598-023-46504-2 (PMC10628244; doi:10.1038/s41598-023-46504-2)
Supplement: Supplementary file 1 — Supplementary Information. [file 41598_2023_46504_MOESM1_ESM.docx]

Article

Toxicity effects of zinc supply on growth revealed by physiological and transcriptomic evidences in sweet potato (*Ipomoea batatas* (L.) Lam)

Yusha Meng ^1, 2†^, Chao Xiang ^1†^, Jinxi Huo ^2^, Shengfa Shen ^1^, Yong Tang ^2^ and Liehong Wu ^1,^*

| ). |
| --- |

^1^ Institute of Crops and Nuclear Technology Utilization, Zhejiang Academy of Agricultural Sciences, Hangzhou 310021, Zhejiang, China

^2^ Key Laboratory of Creative Agriculture, Ministry of Agriculture and Rural Affairs, Zhejiang Academy of Agricultural Sciences, Hangzhou 310021, Zhejiang, China

***** Correspondence: [zwsgsz@zaas.ac.cn](mailto:zwsgsz@zaas.ac.cn)

^†^ These authors contributed equally to this work and share first authorship.

**Supplementary materials:**


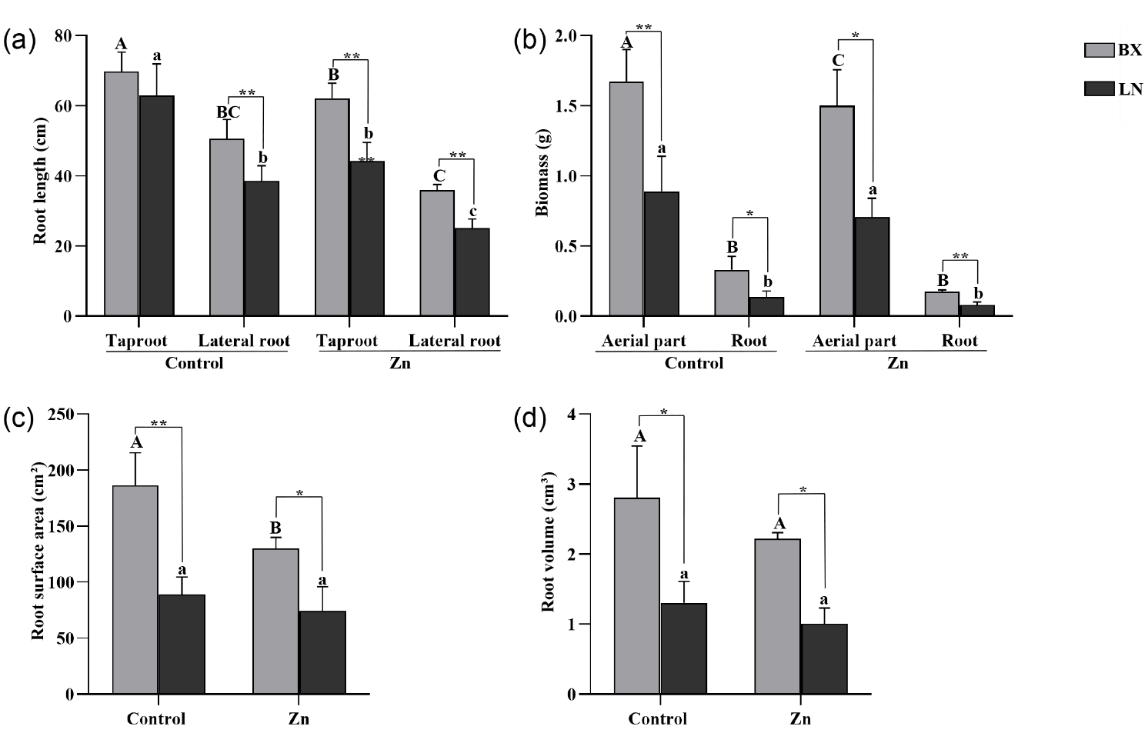


**Figure S1.** Inhibition effects of 40 μM ZnSO_4_·7H_2_O on roots of sweet potato seedlings after 10 days treatment. (a) Effects of excessive Zn on root length. (b) Effects of excessive Zn on biomass. (c) Effects of excessive Zn on root surface area. (d) Effects of excessive Zn on root volume. The lowercase letters indicated the significantly differences between the treatment and the control in LN. The capital letters indicated the significantly differences between the treatment and the control in BX. * and** indicated the significantly differences between BX and LN. BX, Baixinfanshu, LN, Laonangaufanshu. The results were showed as mean ± SD (n = 3), (**P* < 0.05, ***P* < 0.01, Duncan’s test).


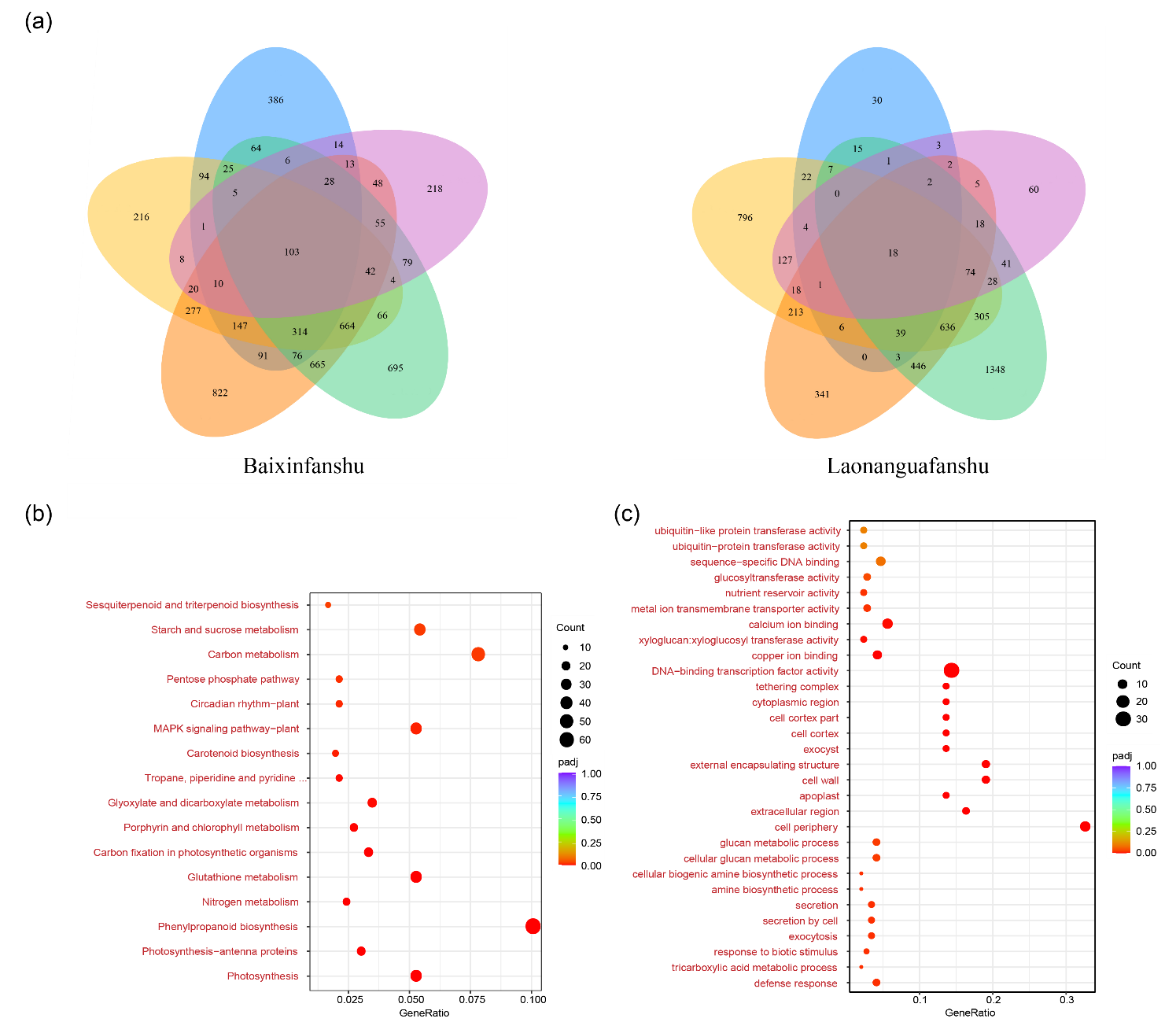


**Figure S2.** (a) Venn diagram of expression patterns of DEGs in different groups. (b) KEGG analysis in 6 H vs. 0 H of BX. (c) GO analysis in 24 H vs. 0 H of LN. BX, Baixinfanshu, LN, Laonangaufanshu.


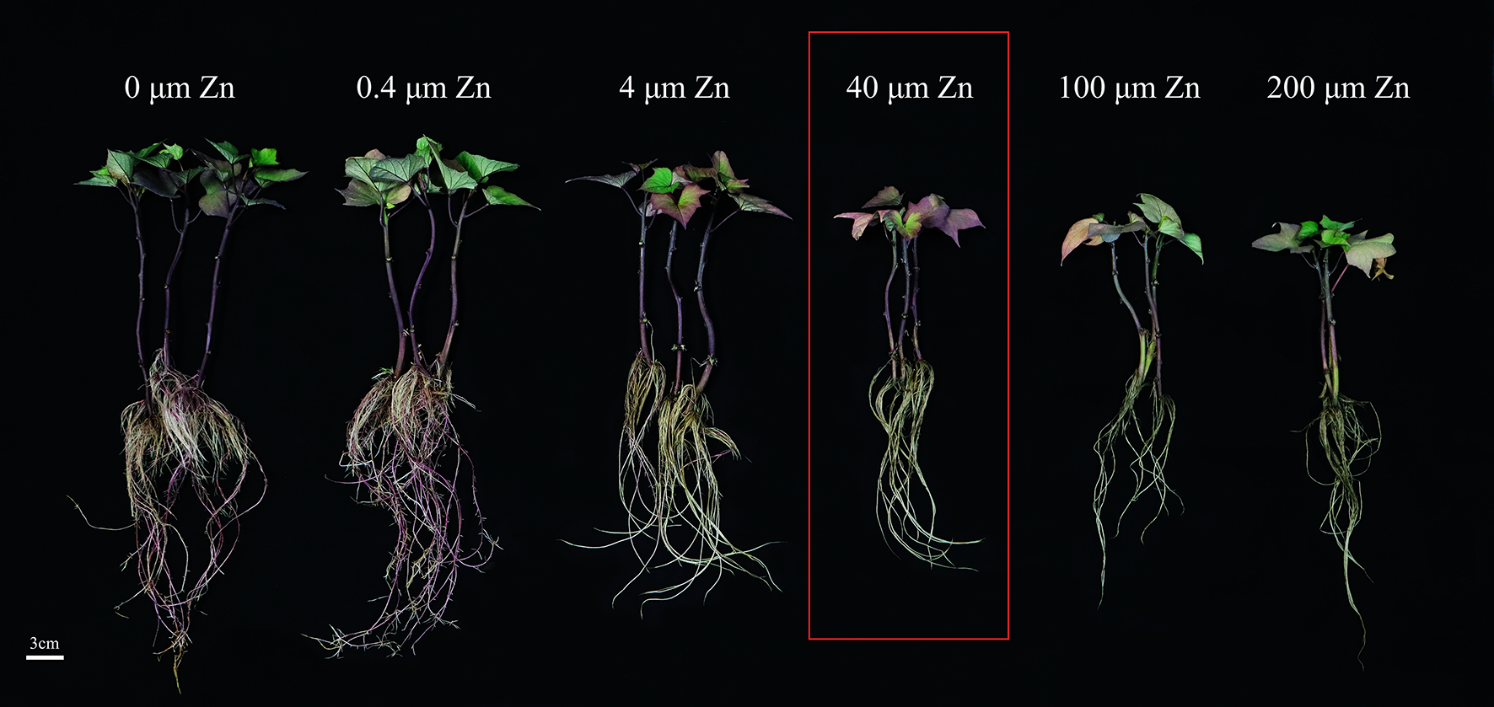


**Figure S3.** Effects of different concentrations of ZnSO_4_·7H_2_O on growth indices after 10 days treatment in sweet potato seedlings. The red box indicates the optimal level of Zn toxicity on sweet potato plants at different levels.

**Table S1.** Uptake and transferring characteristics of Zn under field cultivation

| **Variety** | **Treatment** | **Zn (mg Kg^-1^ DW)** | |
| --- | --- | --- | --- |
|  |  | **Aerial parts** | **storage roots** |
| Baixinfanshu | Initial stage of storage roots enlargement | 15.70±0.18 | 4.31±0.15^A^ |
|  | Harvest stage of storage roots | 10.06±0.77*^a^ | 16.97±0.36**^a^ |
| Laonanguafanshu | Initial stage of storage roots enlargement | 17.6425±1.11 | 7.99±0.54^B^ |
|  | Harvest stage of storage roots | 13.08±0.62**^b^ | 10.24±0.56**^b^ |

**Note:** The lowercase letters indicated the significantly differences in Zn content between the two genotypes sweet potato plants. * and** indicated the significantly differences between the initial stage and the harvest stage. The results were showed as mean±SD (n = 3), (**P* < 0.05, ***P* < 0.01, Duncan’s test).

**Table S2.** Detailed annotation information of DEGs related to 5 known families of Zn transporters

|  | | | | | |
| --- | --- | --- | --- | --- | --- |
| **Family** | **Gene ID** | **Expression pattern** | | **Gene chromosome** | **TF_family** |
|  |  | **Baixinfanshu** | **Laonanguafanshu** |  |  |
| ZIP | itf01g03600 |  | down | Chr01 | START |
|  | itf01g16700 | down | down | Chr01 | bZIP_1 |
|  | itf01g22350 | down | down | Chr01 | bZIP_1 |
|  | itf01g28120 | up | up | Chr01 | - |
|  | itf01g34950 | down | down | Chr01 | bZIP_1 |
|  | itf02g16910 | up |  | Chr02 | - |
|  | itf02g20400 | down | down | Chr02 | Homeobox |
|  | itf03g07840 |  | down | Chr03 | Homeobox |
|  | itf04g04220 | down | down | Chr04 | Homeobox |
|  | itf04g28570 | down |  | Chr04 | - |
|  | itf05g12890 | down | down | Chr05 | bZIP_1 |
|  | itf05g24140 | down | down | Chr05 | bZIP_1 |
|  | itf05g24550 | down | down | Chr05 | Homeobox |
|  | itf05g27440 | down | down | Chr05 | - |
|  | itf06g10300 | down | down | Chr06 | - |
|  | itf06g20310 | down | down | Chr06 | - |
|  | itf06g22880 | down | down | Chr06 | bZIP_1 |
|  | itf07g00390 | down | down | Chr07 | bZIP_1 |
|  | itf07g12880 | down | down | Chr07 | bZIP_1 |
|  | itf07g22640 | down | up | Chr07 | Homeobox |
|  | itf08g07170 | down | down | Chr08 | - |
|  | itf08g07220 | down | down | Chr08 | - |
|  | itf08g15800 | down | down | Chr08 | HALZ |
|  | itf08g16070 | up | down | Chr08 | HALZ |
|  | itf09g01100 | down | down | Chr09 | bZIP_1 |
|  | itf09g01780 | down | down | Chr09 | bZIP_1 |
|  | itf10g12770 | down | down | Chr10 | bZIP_1 |
|  | itf12g03640 |  | down | Chr12 | Homeobox |
|  | itf12g16540 | down | up | Chr12 | Homeobox |
|  | itf12g16550 | down | up | Chr12 | Homeobox |
|  | itf12g18310 | up | up | Chr12 | bZIP_1 |
|  | itf13g22080 | down | down | Chr13 | bZIP_1 |
|  | itf14g02770 | down | down | Chr14 | bZIP_1 |
|  | itf14g12010 | down | down | Chr14 | bZIP_1 |
|  | itf15g02970 |  | down | Chr15 | bZIP_1 |
|  | itf15g19800 | down | down | Chr15 | bZIP_1 |
|  | novel.4265 | up | up | Chr09 | bZIP_1 |
| Natural resistance-associated macrophage protein | itf13g12280 | down | down | Chr13 | - |
| Major facilitator superfamily | itf15g05130 | down | up | Chr15 | MFS_1 |
|  | itf03g13380 | down | down | Chr03 | MFS_1 |
|  | itf03g21270 | down | down | Chr03 | MFS_1 |
|  | itf04g01140 | down |  | Chr04 | MFS_1 |
|  | itf04g04080 |  | down | Chr04 | - |
|  | itf04g30980 | down | up | Chr04 | MFS_1 |
|  | itf05g06020 | up | up | Chr05 | MFS_1 |
|  | itf05g21910 | up |  | Chr05 | MFS_1 |
|  | itf07g11920 | down | up | Chr07 | MFS_1 |
|  | itf07g11930 | down | up | Chr07 | MFS_1 |
|  | itf07g11940 | down | up | Chr07 | MFS_1 |
|  | itf08g13870 | up | up | Chr08 | MFS_1 |
|  | itf09g09080 | up |  | Chr09 | MFS_1 |
|  | itf09g14490 | up |  | Chr09 | MFS_1 |
|  | itf09g14500 | up |  | Chr09 | MFS_1 |
|  | itf11g00450 | down |  | Chr11 | MFS_1 |
|  | itf11g00840 | down | down | Chr11 | MFS_1 |
|  | itf11g07520 | up | up | Chr11 | MFS_1 |
|  | itf11g21330 | down | down | Chr11 | MFS_1 |
|  | itf12g21160 |  | up | Chr12 | MFS_1 |
|  | itf15g05130 | down | up | Chr15 | MFS_1 |
|  | itf15g05140 | down | up | Chr15 | MFS_1 |
| P-type ATP-ase | itf02g06400 |  | down | Chr02 | - |
| Metallothionein, family | novel.5448 |  | up | Chr14 | - |
|  | itf09g08510 | down | down | Chr09 | - |
